# Novel and flexible ultrasound simulation with smartphones and tablets in fetal echocardiography

**DOI:** 10.1007/s00404-021-06102-x

**Published:** 2021-06-04

**Authors:** Tim Johannes Hartmann, Ulrike Friebe-Hoffmann, Nikolaus de Gregorio, Amelie de Gregorio, Christiane Lato, Beate Hüner, Thomas Friedel, Wolfgang Janni, Krisztian Lato

**Affiliations:** Department of Obstetrics and Gynaecology, University Hospital Ulm, Prittwitzstraße 43, 89075 Ulm, Germany

**Keywords:** Ultrasound simulator, Fetal echocardiography, Ultrasound education, Portable ultrasound simulation, Medical education

## Abstract

**Purpose:**

Evaluation of a novel ultrasound-simulation-app for training fetal echocardiography as a possible useful addition for students, residents and specialist doctors. Furthermore, comparison to a conventional learning-method with special attention on orientation and recognition of physiological structures.

**Methods:**

Prospective two-arm study with the participation of 226 clinical students. 108 students were given an extract from a textbook on fetal echocardiography (PDF-group, *n* = 108) for 30 min to study. 118 students were able to use the new ultrasound-simulator-app (Simulator-group, *n* = 118) to learn for 30 min. The knowledge of the students was examined both before and after the learning-period by having them identify sonographic structures in videos using single-choice selection.

**Results:**

There were no significant differences between the two groups regarding age (*p* = 0.87), gender (*p* = 0.28), and the number of previously performed ultrasound-examinations (*p* = 0.45). In the Simulator-group, there was a significantly higher learning effect regarding the proportion of students with an increase of correct answers in the video test examination (*p* = 0.005). At the end of learning, the students in the Simulator-group needed significantly less time to display the structures in the app’s simulation (median initially 10.9 s vs. 6.8 s at the end; *p* < 0.001).

**Conclusions:**

The novel ultrasound-simulation-app seems to be a useful addition and improvement to ultrasound training. Previous difficulties such as simultaneously having patients, ultrasound-machines, and professors at disposal can thus be avoided. This means that another important step towards remote learning can be taken, which has been proven increasingly essential lately, due to the COVID-19 pandemic.

## Introduction

Ultrasound examinations are an established diagnostic procedure in medicine. Diagnostic advantages are the non-invasiveness, the brief examination duration, the cost efficiency, and the lack of relevant side effects at high-diagnostic validity in almost all clinical areas of medicine [1]. Ultrasound examinations are an integral part of prenatal care. Due to the relatively high incidence of fetal heart defects and its complexity, fetal echocardiography is of particular importance within the field. Since introducing the “extended basic ultrasound examination” in Germany in 2013, the focus has been inter alia on obtaining the 4-chamber view correctly [2]. The training is implemented using the literature, hands-on-training, courses, and lectures; however, practical expertise can only be acquired to a certain extent through volunteers and impositions additional time-constraints. Furthermore, it is impossible to present pathological findings in healthy volunteers. Recent circumstances have made it nearly impossible for many sonography students worldwide to gain the essential practical hands-on-expertise due to the COVID-19 pandemic. A multitude of programs have limited or suspended their scanning-labs where students can practice with real patients [3, 4].

The significance of the existing ultrasound-simulators has been proven—they are already in frequent use, especially in the Anglo–American region [5–9]. Integrated, didactically structured teaching modules help in the training of both students and doctors while being independent of patients. These modules have been proven to effectively convey knowledge of ultrasound [6, 9, 10]. However, established simulators are often technically complex and bulky devices with additional hardware, mandatory for registering the learner’s movements.

Medical apps are software programs that integrate texts, images, videos, or web-content in a learning platform [11] and help effectively with learning [12–14]. To date, such teaching applications already exist in ultrasound training. However, these often exclusively contain 2-dimensional flashcards and instructional videos. The first mobile 3D ultrasound application for smartphones was published in 2017 [15]. In it, the kidney ultrasound was simulated via augmented reality. In 2018, Scanbooster developed the world’s first realistic ultrasound simulator application for smartphones and tablets, which was examined in this study [16].

In this investigation, we evaluated the benefit of the newly developed Scanbooster ultrasound simulator app. In particular, we analyzed the learning and understanding of the basics of fetal echocardiography. In addition, the app was compared to a conventional learning method, with particular interest in the orientation and recognition of physiological anatomical structures.

## Materials and methods

The presented study is a prospective two-arm comparative study with 226 medical students, who were recruited for the study between April and December 2019 as part of an ultrasound seminar.

Before the study was carried out, the respective participants were informed about the course of the study, its voluntary nature and the absence of any (negative) consequences in the event of non-participation. According to a request to the Ethics Comission in Ulm, this study fell under §15 of the professional code for physicians in Baden-Württemberg, and therefore, did not require an ethics vote.

**Fig. 1:**
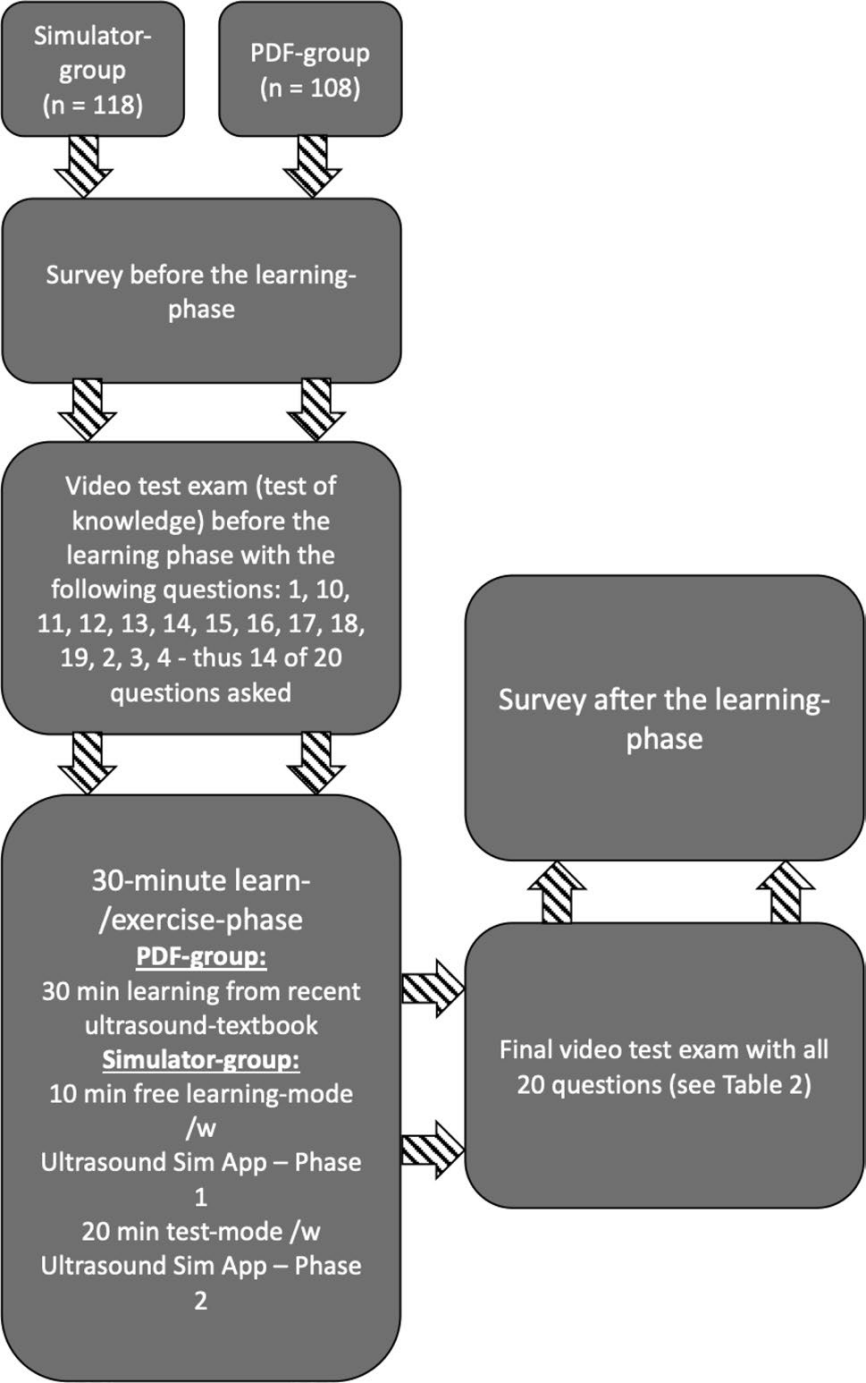
Course of the study

A stand-alone study app was specially developed for the study, in which all parts of the study (including a survey at the beginning, a video test before the learning phase, both different learning methods (PDF document and ultrasound simulator), a video test after the learning phase and a final survey questionnaire) were integrated. The study supervisor thus only had a passive monitoring function. An extended overview of the study’s course is show in Fig. 1.

After starting the study app, the students were automatically randomized into two groups using a program function inside the app [17] with an equal distribution probability-ratio of 50% into either group (PDF-group, *n* = 108; Simulator-group, *n* = 118). The underlying program function was “Bool.random()”, which produces a Boolean value with equal probability[17]. As there are only two possible states of a Boolean value (True and False), the program checked the result and allocated a student to the PDF-group if the result was “True” and to the Simulator-group if it was “False” [17]. Further details on the complex mechanisms used to provide random Boolean values are provided in the documentation of the programming language used (Swift) [17].

### Survey at the beginning

Initially, the students were asked about their age, gender, affinity to technology, experience in technology and ultrasound as well as further characteristics on their previous academic performance, using a questionnaire. The questionnaire was implemented into the study app by allowing single choice answers, multiple choice answers, sliders with the current value being displayed (e.g., the age) and sliders with arbitrary boundaries without a value being displayed during selection (e.g., the boundaries of the question regarding the experience of the participant in ultrasound were “little” to “much”; internally such values were tracked with floating-point precision between 0–1).

### Videotest-exam before the learning-phase

Next, the participants’ previous knowledge baseline with regards to recognition of physiological anatomical structures in ultrasound was tested to later determine the students’ difference in knowledge after the intervention, if any. This test was performed by the use of short ultrasound videos in which the respective structure was marked with a yellow “X” that moved along with the structure, if the structure moved during the course of the video. The participants had to indicate which of five given possible structures was marked with the yellow “X” in the short video. A total of 14 such video questions was asked in the same sequence for all participants before the learning phase (according to Table 1, the sequence was: 1, 10, 11, 12, 13, 14, 15, 16, 17, 18, 19, 2, 3, 4).

**Table 1:** List of the 20 anatomical structures that were presented to the students of both groups in the video test before and after the 30-min learning/practice phase

Index of the question	Structure that was marked in the video	Answer option 1	Answer option 2	Answer option 3	Answer option 4	Answer option 5
0^a^	Aorta	Aorta	Pulmonary trunk	RVOT	A. pulmonalis	V. cava
1^b^	A. mesenterica superior	V. porta hepatis	Aorta	V. splenica	A. mesenterica superior	V. cava inferior
2^b^	Caput pancreatis	Corpus pancreatis	Caput pancreatis	Cauda pancreatis	Spleen	Uncinate process of pancreas
3	Cerebellum	Liver	Kidney	Sinus sagittalis superior	Heart	Cerebellum
4	Femur	Femur	Humerus	Tibia	Fibula	Scapula
5^a^	Heart	Liver	Kidney	Spleen	Heart	Pancreas
6^a^	Left ventricle	Aorta	Left atrium	Right atrium	Left ventricle	Right ventricle
7^a^	Stomach	Vesica biliaris	Kidney	Stomach	Heart	Urinary bladder
8	Kidney	Liver	Kidney	Spleen	Heart	Pancreas
9^a^	Right atrium	Right atrium	Left atrium	Right ventricle	Left ventricle	Pancreas
10^a^	Thymus	Liver	Thymus	Diaphragm	Heart	Pancreas
11^a^	Pulmonary trunk	V. cava inferior	LVOT	Aorta	Pulmonary trunk	A. pulmonalis sinister
12^a,b^	V. cava inferior	V. cava inferior	V. porta hepatis	V. cava superior	V. azygos	A. pancreaticoduodenalis superior
13^b^	V. lienalis	V. hepatis	V. cava inferior	V. splenica	V. lienalis	A. splenica
14^b^	V. porta hepatis	V. splenica	A. mesenterica superior	Aorta	V. porta hepatis	V. cava inferior
15^a^	V. cava superior	V. cava superior	V. cava inferior	Aorta	V. porta hepatis	Ductus arteriosus
16^a^	V. cava inferior	V. cava superior	V. cava inferior	Aorta	V. brachiocephalica dex	Ductus arteriosus
17^a^	Foramen ovale (Botalli)	Right atrium	Left atrium	Foramen ovale (Botalli)	Aorta	V. cava inferior
18^a^	Ductus arteriosus (Botalli)	Right atrium	Left atrium	Foramen ovale (Botalli)	Ductus arteriosus (Botalli)	V. cava inferior
19^a^	Aortic valve	Pulmonary valve	Thymus	Tricuspid valve	Aortic valve	V. cava inferior

Each question was assigned an index starting with 0. This included a total of 20 questions (index 0–19). The second column shows the respective structure that was marked in the video with a yellow “X” and thus the correct answer, the columns right of it show all answer options offered to the participant. Indexes of the questions that contained essential structures for performing fetal echocardiography are marked with “^a^”. Indexes of questions that contained video material from adult patients are marked with “^b^”

### Learning-phase

The course of the study for the two groups only differed in the ensuing learning phase: the study app automatically displayed the respective learning materials depending on the group of the participant: the “PDF-group” was shown an excerpt from a standard textbook on fetal echocardiography, which is described in greater detail below. The “Simulator-group” was able to use the new ultrasound simulator app during the learning phase. Both the simulator app and the PDF-document were fully integrated into the study app.

### PDF-group

#### Description

In the PDF-group, the 108 students were able to learn the basics of general fetal echocardiography over 30 min using an excerpt from a current textbook in the form of a fully scroll- and zoomable integrated PDF. The remaining time was shown equivalent to the app group. When viewing the PDF file, all of the standard viewing functions known from usual PDF-viewer programs/apps were possible, such as scrolling, zooming or marking.

#### Learning material

The learning material used (the PDF document) was a nine-page excerpt (pages 391–399) from the standard work Christof Sohn/Wolfgang Holzgreve: Ultraschall in Gynäkologie und Geburtshilfe [18]. This contained the entire chapter “15.7 Fetale Echokardiografie”. This textbook represents a standard work on sonography within gynecology and obstetrics, including the sub-specialization fetal echocardiography, and was therefore, selected as learning material. The chapter “15.7 Fetale Echokardiografie” contained in the learning material provides a complete overview of the specialist field, and should therefore, suffice for answering the questions used in the video test after the learning phase (the reference here is to the questions on fetal echocardiography, General sonographic questions are of course only insufficiently covered in the chapter).

#### Simulator-group

**Fig. 2:**
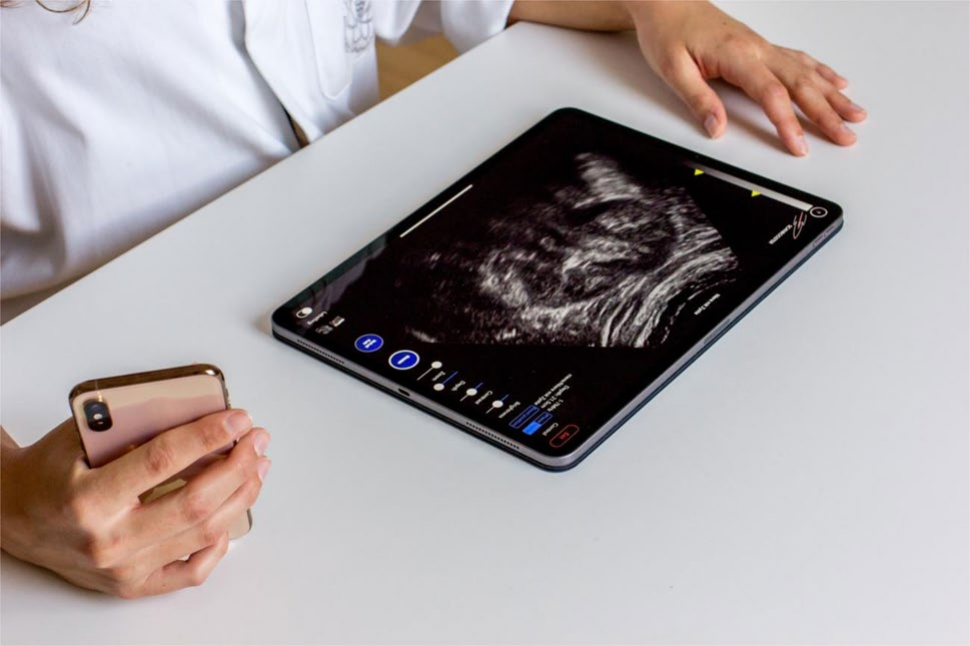
The setup of the simulation. The ultrasound simulation was displayed on a tablet (serving as a display), the control was implemented by a smartphone (serving as a virtual probe) with the Scanbooster Control app installed on it. The two devices were subsequently connected via Bluetooth. By moving the smartphone in space, the corresponding ultrasound image was realistically calculated by the app and displayed on the tablet in real-time

In the Simulator-group, the 118 students were able to learn and practice for 30 min with the new ultrasound simulator app. The ultrasound simulation was displayed on a tablet (serving as a display), the control was implemented by a smartphone (serving as a virtual probe) with the Scanbooster Control app installed on it. The two devices were subsequently connected via Bluetooth. By moving the smartphone in space, the corresponding ultrasound image was realistically calculated by the app and displayed on the tablet in real-time (Fig. 2). The Scanbooster Control App installed on the smartphone registered the following movements: orientation (rotation around the X, Y, and Z axes) and position in space (X, Y, and Z position). The positional data were acquired by the specially developed realScan technology [19]. The simulation of a fetus in the 28th week of pregnancy was displayed on the tablet. Considering the relatively short learning period of 30 min, two auxiliary features were integrated into the app: 1.) The students in the Simulator-group could restore the initial position (standard 4-chamber view) at any time by pressing the “Reset Position” button and proceed with their examination from there. 2.) Only rotations (rotation around the X, Y, and Z axes) were enabled for the students in the control. This served to both disable loss of contact simulation with the possibly resulting “black image” and prevent possible movement (drifting) away from the sonographic area of interest.

**Fig. 3:**
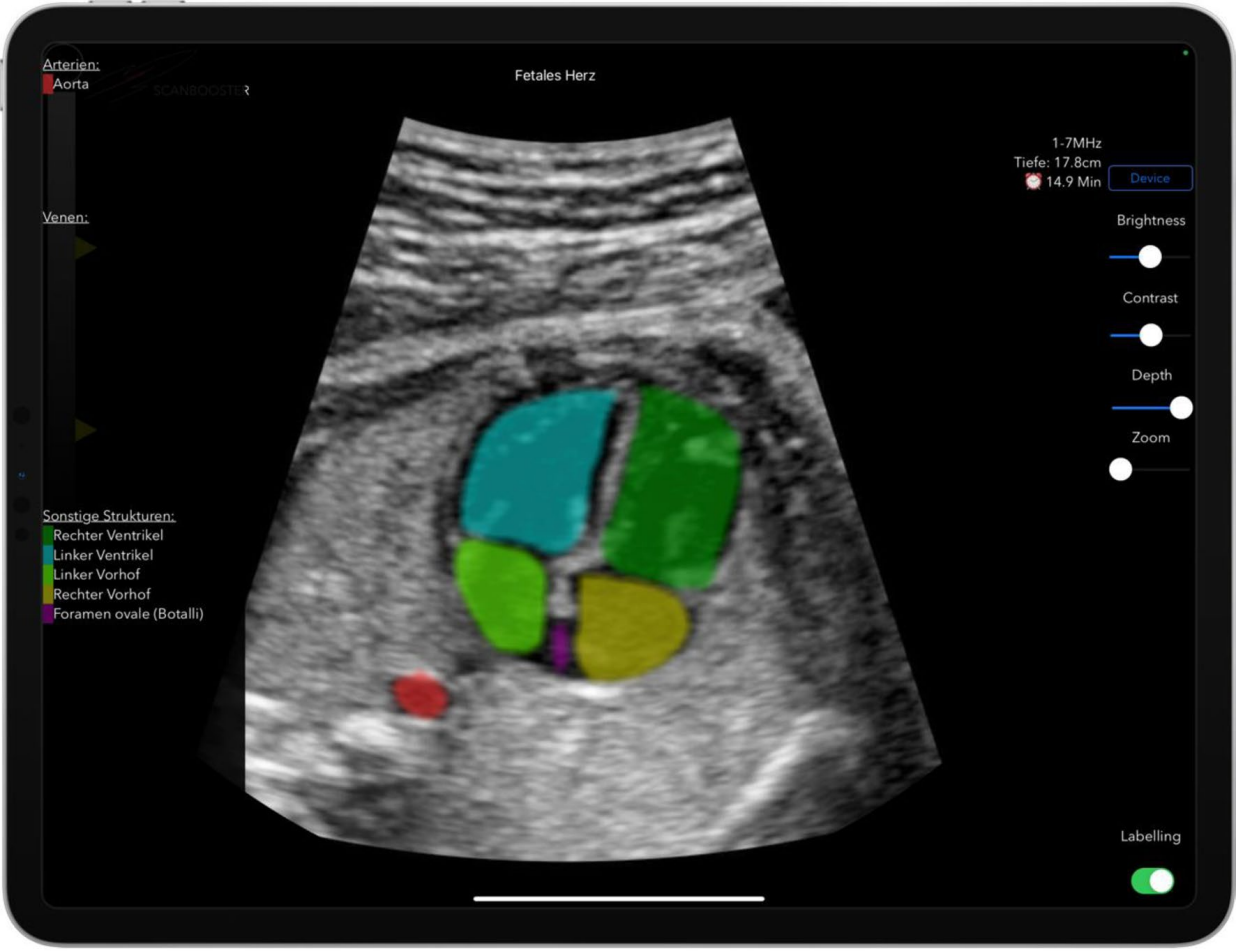
The ultrasound simulation with enabled labeling. During the free learning phase (phase 1—learning mode), the anatomical structures could be labeled with color in real time by clicking on the “Labeling on” button (shown in the lower right area of the image) and overlaid with an automatically adapting legend (shown in the left image area). Displayed with enabled labeling

**Fig. 4:**
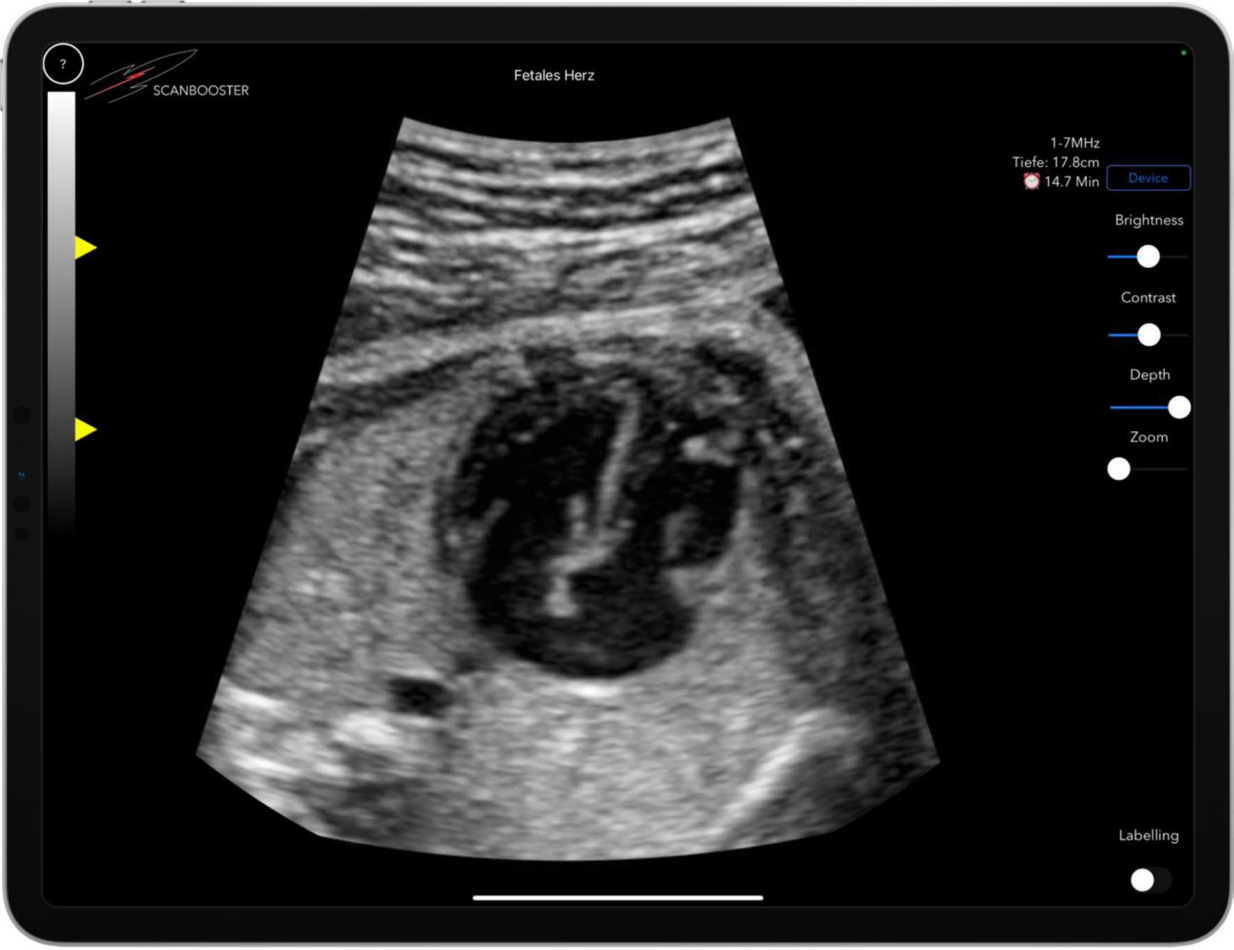
The ultrasound simulation with disabled labeling. During the free learning phase (phase 1—learning mode), the anatomical structures could be labeled with color in real time by clicking on the “Labeling on” button (shown in the lower right area of the image) and overlaid with an automatically adapting legend. Displayed with disabled labeling

In the first 10 min (phase 1—learning mode) the students of the Simulator-group were able to freely scan with the simulation. By pressing the “Reset Position” button, the position of the virtual transducer was set as a new reference and the initial position and orientation were thus displayed. This was the standard 4-chamber view. By fanning cranially with the virtual transducer, the 5-chamber view, the 3-vessel view, and a total of 19 anatomical structures (see Table 2) could be displayed on the tablet. In this learning phase, the anatomical structures could be highlighted with color and overlaid with an automatically adapting legend in real-time by clicking on the “Labeling on” button (Fig. 3, 4).

**Table 2:** List of 19 anatomical structures that were presented to the students of the Simulator-group in the 30-min learning/practice phase with the ultrasound simulator app (phase 1: 10 min learning mode; phase 2: 20 min practice mode)

Structure
Aorta
Atrium cordis dextrum
Atrium cordis sinistrum
Ductus arteriosus
Ductus venosus
Foramen ovale
Sinus portalis
Thymus
Trachea
Truncus pulmonalis
Vena brachiocephalica dextra
Vena brachiocephalica sinistra
Vena cava inferior
Vena cava superior
Vena umbilicalis
Ventriculus (gaster)
Ventriculus cordis dexter
Ventriculus cordis sinister
Vesica biliaris

The 10-min learning phase was followed by a 20-min practice phase (phase 2—practice mode). The labeling function was automatically deactivated. All the integrated anatomical structures were chosen randomly by the app and initially had to be displayed in the simulation by the students. If the student had supposedly correctly displayed the structure he was looking for, he had to confirm his selection by pressing the “Freeze” button (the simulation was thereby stopped). The student then received feedback: If the requested structure was displayed incorrectly, the app offered help (the possibility to switch on the labeling despite deactivation), else a popup was shown, indicating the correct display of the structure. As soon as the structure was correctly displayed, the student had to touch its exact location in the image. This should prevent the structure from being displayed by chance. If there were more than four unsuccessful attempts to touch the structure, the app offered to display the wanted structure by means of a colored overlay. If a searched structure was correctly displayed and touched on the first attempt, this structure was not queried again for the moment. If all structures were correctly displayed and touched without help at least once, all structures were queried again. Structures that caused the students difficulties were thus queried and practiced more often than those that were shown correctly on initial attempt.

#### Videotest-exam after the learning-phase

The ability of all students to correctly identify physiological anatomical structures in ultrasound videos was examined once again, using the same setup as already used before start of the learning phase: Of the 20 video questions (see Table 1), the 14 questions, that had already been asked before the learning phase were asked again in the same sequence, followed by the remaining six new questions. This setup was chosen to measure the impact of re-asking a question and subsequently determine the portion of the knowledge gain that can be attributed to the actual learning and that, attributable to asking the question again. Altogether, not only structures with importance for fetal echocardiography but also structures of prenatal ultrasound and general adult ultrasound were tested: 13 questions showed structures that are essential for performing fetal echocardiography; whereas, seven questions showed structures that are not. Structures, virtually irrelevant for fetal echocardiography were included as well because a broad overview on the participants’ knowledge of ultrasound in general was to be determined. Furthermore, the effect of using either learning method on non-affected subjects/sonographic structures was to be determined as well. The results for the structures relevant for fetal echocardiography only were evaluated separately. A detailed listing of the videos and contained structures is given in Table 1.

#### Survey at the end

At the end of the study, the students were asked about their impressions, satisfaction, whether they’d recommend their respective learning material and if they would install the app on their own device, had they used the app. The same setup as in the beginning of the study was used for this questionnaire.

### Statistical analysis

The statistics were carried out by specifying absolute and relative frequencies for categorical data as well as median and range for ordinal scaled and non-normally distributed metric data. Group comparisons between Simulator-group and PDF-group with regard to age, semester, percentages, and all data collected using the digital slider were carried out with the non-parametric Mann–Whitney *U* test for independent groups; Group comparisons with regard to categorical data and rates or proportions were made using Chi-squared tests. The pairwise comparison of the times required for a correct representation of the anatomical structures at the beginning and at the end of the exercise phase for students in the Simulator-group was carried out with the non-parametric Wilcoxon matched-pairs signed-ranks test for dependent data. The significance criterion was set to *α* = 0.05; there was no adjustment of the significance level for multiple testing. All *p* values shown are bilateral. The statistics program IBM SPSS Statistics for Windows, Version 25 (Armonk, NY: IBM Corp.) was used for all statistical analyses.

## Results

### Characteristics and previous knowledge of the participants

There were no significant differences between the Simulator-group and the PDF-group with regard to the parameters age (*p* = 0.87) and gender (*p* = 0.28). According to their own assessment in the survey before the learning phase, the students in the Simulator-group had a significantly higher affinity to technology (scale from 0 to 100; median 60.5 vs. 50.0; *p* = 0.002) and a significantly higher technical experience (scale from 0 to 100; median 39.5 vs. 26.6; *p* = 0.008) than the students in the PDF-group. While their own assessment of the experience in the field of ultrasound tended to be slightly higher among the students in the Simulator-group than among the students in the PDF-group (scale from 0 to 100; 14.6 vs. 11.6; *p* = 0.076), there were no differences in terms of experience regarding the number of previously performed ultrasound examinations (*p* = 0.445; see Table 3).

**Table 3:** Characteristics and previous knowledge of the participants

Variable	PDF-group *N* = 108	Simulator-group *N* = 118	*p* value
Age (years)			0.872^a^
Median	24.0	24.0	
Range	19–33	21–40	
Sex			0.278^b^
Male	47 (43.5%)	43 (36.4%)	
Female	61 (56.5%)	75 (63.6%)	
Semester of the medical student			0.091^a^
Median	9	9	
Range	7–13	7–12	
Affinity to technology (scale 0–100)			0.002^a^
Median	50.0	60.5	
Range	0.0–100.0	0.0–100.0	
General experience with technology (scale 0–100)			0.008^a^
Median	26.6	39.5	
Range	0.0–83.7	0.0–100.0	
General experience in ultrasound (scale 0–100)			0.079^a^
Median	11.6	14.6	
Range	0.7–60.6	0.0–77.2	
Number of previously performed sonographic examinations			0.445^b^
None	0 (0.0%)	0 (0.0%)	
1–5	26 (24.1%)	27 (22.9%)	
6–10	37 (34.3%)	47 (39.8%)	
11–20	28 (25.9%)	21 (17.8%)	
> 20	17 (15.7%)	23 (19.5%)	

^a^Mann–Whitney *U* test

^b^Chi-squared test

### Results of the Simulator-group and PDF-group in the video test before and after the 30-min learning/practice phase (questions about all anatomical structures)

**Fig. 5:**
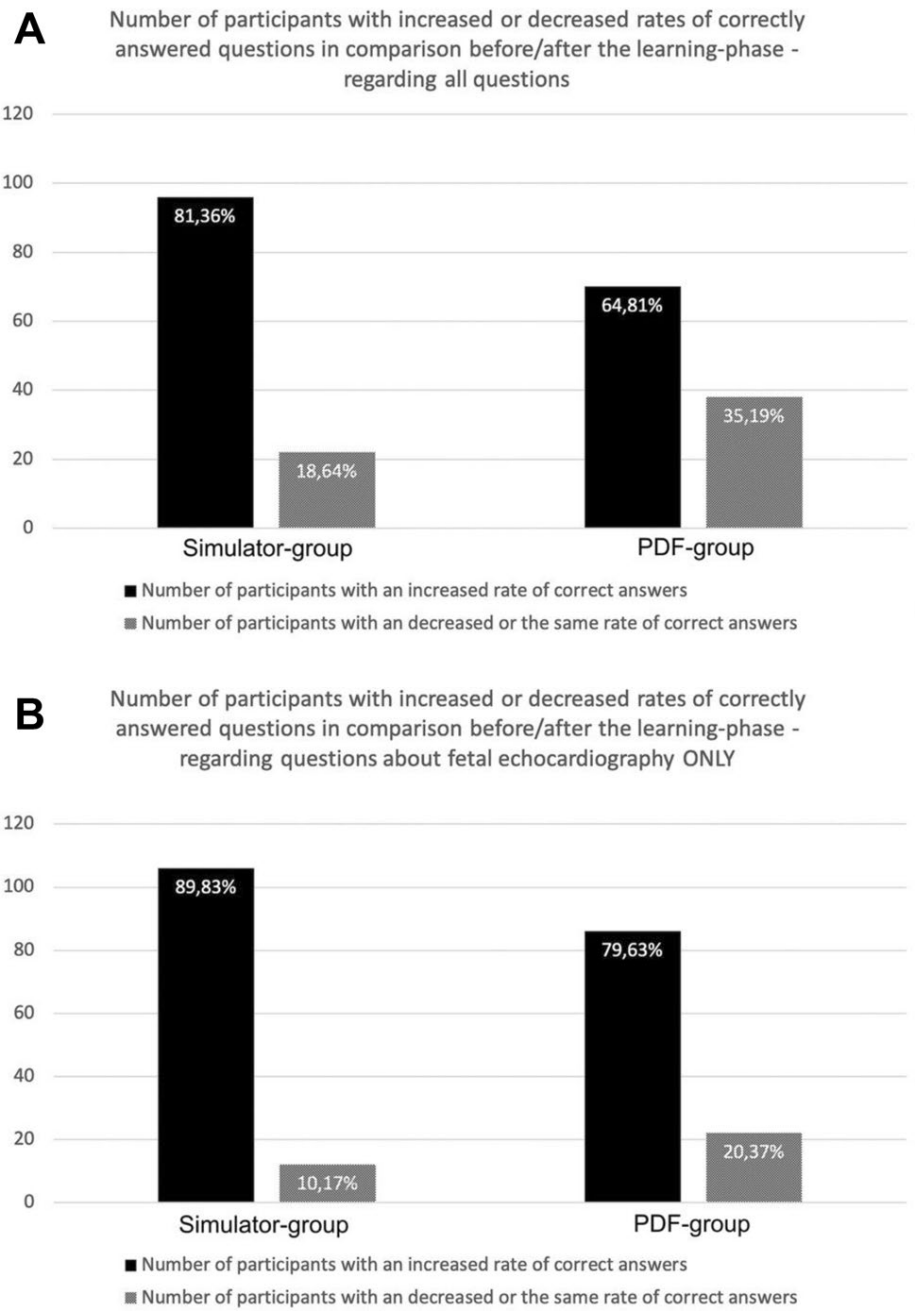
Statistics of the learning-effect. Comparison of the learning effect between the Simulator-group and the PDF-group: number and proportion of users from each group that showed either a positive learning rate or a negative/the same learning rate (measured by the rate of correct answers in the video exam before the learning phase vs. after the learning phase). **a** examined were all questions of the video test exam (*p* = 0.005). **b** examined were only questions related to fetal echocardiography (*p* = 0.032)

Before the learning phase, there was no significant difference between the Simulator-group and the PDF-group in terms of the rate of correctly answered questions in the video test exam (Simulator-group: median 50.0%, range 14.3–85.7%; PDF-group: median 50.0%, range 21.4–85.7%; *p* = 0.421). In contrast, the students in the Simulator-group were able to correctly answer a significantly higher proportion of the questions in the final video test after the practice phase than the students in the PDF-group (Simulator-group: median 65.0%, range 30.0–90.0%; PDF-group: median 55.0%, range 20.0–85.0%; *p* < 0.001). As a result, the Simulator-group’s participants showed a significantly higher learning effect as well, measured by the proportion of participants with an increased rate of correct answers in the final video test. In the Simulator-group, 96 (81.4%) participants showed an increased rate of correct answers, comparing the rates of the initial and final video test, while in the PDF-group this was true only for 70 (64.8%) participants (*p* = 0.005; Fig. 5A).

The positive learning effect in the Simulator-group was already evident during the 20-min exercise phase (phase 2-exercise mode), during which the time required to correctly display the anatomical structures in the simulation decreased significantly (start of the exercise phase: median 10.9 s, range 0.1–218.5 s; end of the exercise phase: median 6.8 s, range 0.1–337.0 s; *p* < 0.001).

### Results of the Simulator-group and PDF-group in the video test before and after the 30-min learning-phase (questions about anatomical structures from the field of fetal echocardiography)

The Participants did not have significant prior knowledge in the area of fetal echocardiography (Simulator-group: median 35.7%, range 0.0–85.7%; PDF-group: median 28.6%, range 0.0–85.7%; *p* = 0.494).

The Simulator-group and the PDF-group did not differ significantly in terms of the proportion of correctly answered questions in the video test before the 30-min learning phase. After the learning phase, the students in the Simulator-group showed a significantly higher rate of correctly answered questions than the students in the PDF-group (Simulator-group: median 63.6%, range 27.3–100.0%; PDF-group: median 54.5%, range 18.2–100.0%; *p* < 0.001). The significantly higher learning effect in the Simulator-group showed again in relation to the proportion of students with an increased rate of correct answers in the final video test examination after the learning/practice phase (Simulator-group: 106 (89.8%); PDF-group: 86 (79.6%; *p* = 0.032; see Fig. 5B).

### Subjective impressions and satisfaction at the end of the study

The students in the Simulator-group had significantly more fun learning with the ultrasound simulation app than the students in the PDF-group with conventional learning (scale from 0 to 100; Simulator-group: median 68.2, range 0.0–100.0; PDF-group: median 10.2, range 0.0–70.0; *p* < 0.001). The subjectively perceived learning effect was also significantly higher in the Simulator-group (scale from 0 to 100; Simulator-group: median 68.9, range 6.9–100.0; PDF-group: median 15.3, range 0.0–88.8; *p* < 0.001).

In the Simulator-group, 93 (78.8%) of the 118 students would prefer ultrasound training using an app to the conventional learning method, whereas no significant differences in gender, technology affinity, and ultrasound-knowledge have been observed between the students who answered this question with “yes” or “no” (all *p* > 0.2). In addition, 116 (98.3%) of the students in the Simulator-group saw the app as a useful addition to conventional learning. A total of 115 (97.5%) students in the Simulator-group said they would download the app on their own device; 106 (89.6%) students would recommend the use of it.

## Discussion

In this study, we did not want to question the validity of the conventional teaching material, but rather evaluate the use and usability of the novel ultrasound-simulation-app for training fetal echocardiography. In detail, we wanted to determine whether it could be a possible, useful addition to the pre-existing training and further education of students, residents and specialist doctors. We wanted to achieve this by comparison to a conventional learning-method with special attention on orientation and recognition of physiological structures.

This first pilot study has proven that the newly developed ultrasound simulator app for smartphones and tablets can help with learning about complex fetal structures. During the use of the app, a significant improvement in the correct display of structures, and in the subsequent video test, a significant increase in the performance of the correct recognition of anatomical, especially cardiac structures, was shown. The built-in real-time labeling (colored overlay of the structures) made it easier to recognize anatomical structures and the continuous challenge (e.g., through the exam mode) apparently also had a motivating effect, as the students in the Simulator-group subjectively had much more fun while learning than the participants in the PDF-group. A smartphone and tablet-based ultrasound app could soon offer far-reaching possibilities: To enable anyone to practice ultrasound, independently of place, time, availability of patients or teaching staff. Neither count nor variety of integrable, fully scannable cases are limited, nor is the variety of medical specialities.

The study results suggest that the integrated exercise mode, in which a structure is queried until it has been correctly set at least once, leads to a highly effective increase in knowledge. After all, structures that are subjectively difficult for the user are asked more often than trivial, initially correctly displayed structures. Overall, the app seems to be not only effective but also user-friendly: most students would prefer the ultrasound simulation app to the conventional learning method. In addition, no significant differences in gender, technology affinity, and ultrasound-knowledge have been observed between the students who did answer to prefer the app to the conventional learning method and those who did not prefer the app, which implicates that the app is well usable for virtually anyone: Both users completely inexperienced in ultrasound and these who already have some experience, even users who possess little or no affinity to technology.

Further follow-up studies have already started to test the effectiveness of a combination of conventional and app-based knowledge transfer.

## Limitations

Besides the small number of participants in the pilot study, another limitation was the short effective learning time: the seminar was subject to a time limit of 60 min. After subtracting the introduction, questioning, and test phase (before and after the practice phase), only 30 min remained for effective learning. Since the students had no prior knowledge of fetal echocardiography, the requirements for exploring the volume and the associated anatomical structures were optimized for the Simulator-group by including a “reset” button and only enabling reduced degrees of freedom in the control (see Methods section). Another limitation of the study was the size of the built-in ultrasound volume and the associated limited field of sonography (organs from head to urinary bladder were included, but the fetal extremities were not available). However, the app also allows display or integration of significantly larger volumes.

Only with regard to the affinity for technology and previous experience with technology and the question of whether video games were or are being played earlier or currently, the app group can be said to have an advantage over its comparison group. This is an important bias that limits the meaningfulness of the learning effects and at the same time means that the app group has an advantage in terms of operating not only their learning part (the ultrasound simulation), but also the entire study app, as a certain technical understanding is required for operating any app. However, it should be noted here that the operation of the app and the correct setting of the structures are to be assessed as more difficult than scrolling through a PDF document or pressing buttons during the video test. Thus, the app group would have had a hypothetical advantage that—if the study had been carried out with complementary group assignment—a (slightly) worse result would have been expectable for the app group, since the operation of the simulation is more complex than the navigation through the PDF document. In addition, it should be noted that the level of basic technical understanding assumed to be essential for operating the study app was very likely among all participants, despite their group assignment and/or technical affinity: In times of widespread use of smartphones and thus inevitably also apps, it is hardly tenable that a substantial part of a student population does not understand how to operate them.

### Advantages of the method

The new ultrasound simulation app enables integration of any ultrasound recording in different areas. Physiological and pathological cases alike can be loaded into the app and allow realistic demonstration of malformations and physiological anatomy. As all integrated cases are fully and realistically scannable, users can be prepared for even very rare cases beforehand. This allows students to improve their recognition of basic physiological structures, residents to improve their knowledge of characteristics and their recognition of pathologies and specialists to stay up-to-date and have a look at reference scans that may feature very rare diseases. The new, powerful processors of current and future smartphone generations additionally will allow the integration of 4D ultrasound recordings (pumping heart or blood vessels), which possibly lead to a further improvement in the simulation. A cloud-based online platform has already been created where students, medical assistants and specialists can download various cases and use their own tablets and smartphones to scan themselves. The live-labeling that shows important structures colored in real-time and the exam mode lead to effective learning with success in knowledge and a high fun factor. The new ultrasound app has a high potential for performing certified online exams in the near future. Further follow-up studies have already started to test the effectiveness of a combination of conventional and app-based knowledge gain. Ongoing development of the app is taking place to bring it to computers as well, allowing the use on as many platforms and for as many learners as possible.

## Conclusions

The first results indicate that with the new ultrasound simulator app the representation of complex sonographic structures can be better understood and carried out than with conventional learning methods. This proves it to be a useful addition and improvement to ultrasound training. Previous difficulties such as having patients, ultrasound devices, highly expensive ultrasound-simulators, and teachers available simultaneously, therefore, become irrelevant almost completely and open up a new way of training. Because the learning can take place autonomously with the integrated learning/practice modules and because pre-existing devices (e.g. the students’ own smartphones and tablets) can be utilized, new cost-effective, time-, and personnel-efficient possibilities arise—even for large groups. Further development is in progress to add additional optimization of the technology and enhance the learning experience.

The current events in the context of the COVID-19 pandemic have impressively revealed the problem of acquiring ultrasound experience through direct patient contact: it is to be expected that infection protection will continue to limit the examination of patients for hands-on teaching and training purposes in the future. It will therefore be all the more important in the future to develop efficient and realistic simulation methods to ensure qualified medical training and advanced education.

## Data Availability

The datasets generated during and/or analyzed during the current study are available from the corresponding author on reasonable request.

## Code availability

The source code of the study app/simulator app is not available due to third party copyrights.
